# Design of Improved Intertrochanteric Fracture Treatment (DRIFT) Study: Protocol for Biomechanical Testing and Finite Element Analysis of Stable and Unstable Intertrochanteric Fractures Treated With Intramedullary Nailing or Dynamic Compression Screw

**DOI:** 10.2196/12845

**Published:** 2019-07-18

**Authors:** Andreas Panagopoulos, Georgios Kyriakopoulos, Georgios Anastopoulos, Panagiotis Megas, Stavros K Kourkoulis

**Affiliations:** 1 Orthopaedic Department Patras University Hospital Patras-Rio Greece; 2 First Department of Trauma and Orthopaedics General Hospital of Athens Athens Greece; 3 National Technical University of Athens Athens Greece

**Keywords:** trochanteric fractures, cut-out, biomechanical testing, finite element analysis, new implant design

## Abstract

**Background:**

Intertrochanteric hip fractures rank in the top 10 of all impairments worldwide in terms of loss in disability-adjusted years for people aged older than 60 years. The type of surgery is usually carried out with dynamic hip screw (DHS) devices or cephalomedullary nails (CMN). Cut-out of the hip screw is considered the most frequent mechanical failure for all implants with an estimated incidence ranging from 2% to 16.5%; this entails both enhancing our understanding of the prognostic factors of cut-out and improving all aspects of intertrochanteric fracture treatment.

**Objective:**

The Design of Improved Intertrochanteric Fracture Treatment (DRIFT) study’s main objective is to provide intertrochanteric fracture treatment expertise, requirements and specifications, clinical relevance, and validation to improve treatment outcomes by developing a universal algorithm for designing patient- and fracture-oriented treatment. The hypothesis to be tested is that a more valgus reduction angle and implants of higher angles will lead to a more favorable biomechanical environment for fracture healing—that is, higher compressive loads at the fracture site with lower shear loads at the hip screw femoral head interface. A new implant with enhanced biomechanical and technical characteristics will be designed and fabricated; in addition, an integrated design and optimization platform based on computer-aided design tools and topology optimization modules will be developed.

**Methods:**

To test this hypothesis, a biomechanical study comprising experimental loading of synthetic femora (Sawbones Inc) and finite element analysis (FEA) will be conducted. Detailed FEA of existing implants (DHS and CMN) implemented in different clinical cases under walking conditions will be performed to derive the stress and strain fields developed at the implant-bone system and identify critical scenarios that could lead to failure of therapy. These models would be validated against instrumented mechanical tests using strain gages and a digital image correlation process.

**Results:**

After testing, geometric drawbacks of existing implants will be fully recognized, and geometric characteristics will be correlated with critical failure scenarios. The last step would be the numeric design, computer-aided design (using FEA codes and design packages), and optimization of the new proposed implant with regard to improved biomechanical surgical technique and enhanced mechanical performance that will reduce the possibility for critical failure scenarios.

**Conclusions:**

The optimization of the biomechanical behavior of the fracture-osteosynthesis model by the application of the ideal reduction angle and implant is expected to have a positive effect to the rate of mechanical failure and, subsequently, the healing rates, morbidity, and mortality in this fragile patient group.

**International Registered Report Identifier (IRRID):**

DERR1-10.2196/12845

## Introduction

### Background

Hip fractures rank in the top 10 of impairments worldwide in terms of loss in disability-adjusted years for people aged older than 60 years [1]. The absolute number of hip fracture hospitalizations in the United States is estimated to approach 289,000 in 2030 [2], and the global number of hip fractures is expected to increase from 1.26 million in 1990 to 4.5 million by the year 2050 [3]. The estimated cost of treatment in the United States was approximately $10.3 to $15.2 billion per year in 1990 [4] and $17 billion in 2002 [5]. In a recent study [6], the incidence of fractures of the hip in Northern Ireland rose from 54 in 100,000 in 2000 to 86 in 100,000 in 2015; the authors predict an increase to 128 in 100,000 in 2030 if this trend continues. In the United Kingdom, there is an ongoing age-standardized decrease in the rate of hip fractures of 0.5% per year, but it is estimated that the annual incidence will double in the next 25 years [7]. The consequences of hip fractures in elderly individuals are significant in terms of lives lost and associated negative impacts on hip fracture patients’ functioning and quality of life. Even with an integrated, multidisciplinary model for the treatment of hip fragility fractures (90% of operations performed within 48 hours), the in-hospital mortality rate was 2.4% and the overall mortality at 1 year from the intervention 18.7%; full mobility status or a low impairment of mobility was reached in 32.1% of patients [8].

The vast majority of intertrochanteric fractures require surgical repair to withstand the early mobilization and weight bearing required to prevent complications due to prolonged bed rest and aid in fracture healing. The type of surgery is generally based on fracture pattern and patient characteristics and is usually performed with dynamic hip screw (DHS) devices or cephalomedullary nails—proximal femoral nails, proximal femoral nail–antirotation nails, gamma nails, or other implants [9-11]. Since the 1960s, the DHS has become the standard implant for surgical treatment of intertrochanteric fractures as it allows controlled fracture compression [12,13]. Despite additional modifications, such as trochanteric support plates and antirotational screws, unstable fractures are less successfully treated by this method [9,14]. Cephalomedullary nails can provide better lateral wall support in more complex fracture patterns, but cut-out of the hip screw has been described as the most frequent mechanical failure for all implants [15-20].

Cut-out is defined as “the collapse of the neck-shaft angle into varus, leading to extrusion of the screw from the femoral head” [15,16]. Several studies have shown that the incidence of cut-out for different compression hip screws and cephalomedullary nails ranges from 0 to 16.5% [12,15-21] and, in older studies [22-25], even up to 20%. Recent developments including plates, antirotational screws, and cement-augmented fixation techniques indicate that the problem of fixation failure is still unresolved [26-27]. This complication is a multifactorial event affected by a number of variables including patient age and sex, bone quality, fracture pattern, quality of reduction, implant design, and meticulous surgical technique [17,18]. In a recent study by Bojan et al [28], the primary cut-out rate of a gamma nail in 3066 consecutive patients was 1.85% and was strongly associated with unstable fractures involving the trochanteric or cervical regions or both as well as nonanatomical reduction or nonoptimal screw position, which are the only two factors that can be controlled by the surgeon. We therefore believe that further elucidation of the effect of surgical technique on the biomechanical behavior of the fracture after fixation is required, especially concerning the effect of the implant angle, positioning, and reduction angle.

### Study Hypothesis and Aims

The main objective of the Design of Improved Intertrochanteric Fracture Treatment (DRIFT) study is to provide pertrochanteric fracture treatment expertise, requirements and specifications, clinical relevance, and validation to improve intertrochanteric fracture treatment outcomes by designing, fabricating, and verifying an implant with optimized biomechanical performance and surgical technique and develop a universal algorithm for designing patient- and fracture-oriented treatment. The specific technical objectives are as follows:

- Improve the understanding of the factors associated with mechanical failure and the impact of design features of implants currently in use on the biomechanical behavior of the implant-bone interface, provided by orthopedic departments.
- Create numerical methods for the prediction of failure of the bone-implant system under static and fatigue loading conditions. Mechanical tests representing exact geometries of the bone-implant system and applying realistic static and fatigue loading conditions will be designed and executed for the verification of the proposed designs.
- Develop an algorithm for designing patient- and fracture-oriented surgical treatment based on existing and novel implants aiming to minimize mechanical failure incidences.
- Design and fabricate a new implant with enhanced biomechanical and technical characteristics. An integrated design and optimization platform based on computer-aided design tools and topology optimization modules will be developed.

To test these hypotheses, a biomechanical study comprising experimental loading and finite element analysis (FEA) will be conducted.

## Methods

### Ethical Approval

As this is a biomechanical study, no institutional board approval is necessary.

### Biomechanical Testing

The experimental part of the study will be undertaken in the Material Testing Laboratory at the National Technical University of Athens.

#### Femoral Preparation

A minimum of fifteen synthetic femora (Sawbones Inc) of medium size and normal (135°) neck shaft angle will be used. All femurs will contain a polyurethane foam filling of 12.5 pounds per cubic foot density to stimulate the material properties of osteoporotic cancellous bone. Concurrently, the digital image files (Initial Graphics Exchange Specification and Solidworks formats) of these femurs will be procured to ensure accurate modeling of the complex femoral geometry and minimal discrepancies between the experimental data and the subsequent FEA models [29-31].

The implants tested in the experimental study will be the Gamma3 nail cephalomedullary system (Stryker) and the DHS plate-hip screw system (Depuy-Synthes). There will be 2 Sawbones for each implant and fracture configuration, namely 2 Sawbones for stable fractures with gamma nail and another two for each of the following configurations: unstable fracture statically locked with normal tip to apex distance (TAD), unstable fracture statically locked with increased TAD, unstable fracture dynamically locked with normal TAD, and unstable fracture unlocked with normal TAD; likewise with the DHS, there will be testing of stable fracture and unstable fracture with two Sawbones in each category. An additional intact femur will be used to standardize the process and facilitate the FEA model validation; thus, a total of fifteen Sawbones will be used. The implants that will be used in the biomechanical testing would be the Gamma3 nail (180 mm length, 11 mm diameter, and 130° angle, titanium) and DHS (135°, 4-hole plate, steel). Although recent biomechanical data have shown equivalence of 2- and 4-hole plates, clinical data such as the study from Baird et al [32] suggest a possible higher rate of failure in unstable fractures with the 2-hole plate. As the study will involve unstable fractures, we decided to use the 4-hole side plate to reduce confounding factors. Despite the commercial availability of titanium plates for the DHS system, the vast majority of implants used are used with stainless steel plates, which is reflected in our choice of material. The choice of a short nail rather than a long one is reflecting the common practice in the fracture types studied, given that we are not going to study reverse obliquity subtypes or subtrochanteric fractures, where long nails have a clear advantage over short nails.

**Figure 1:**
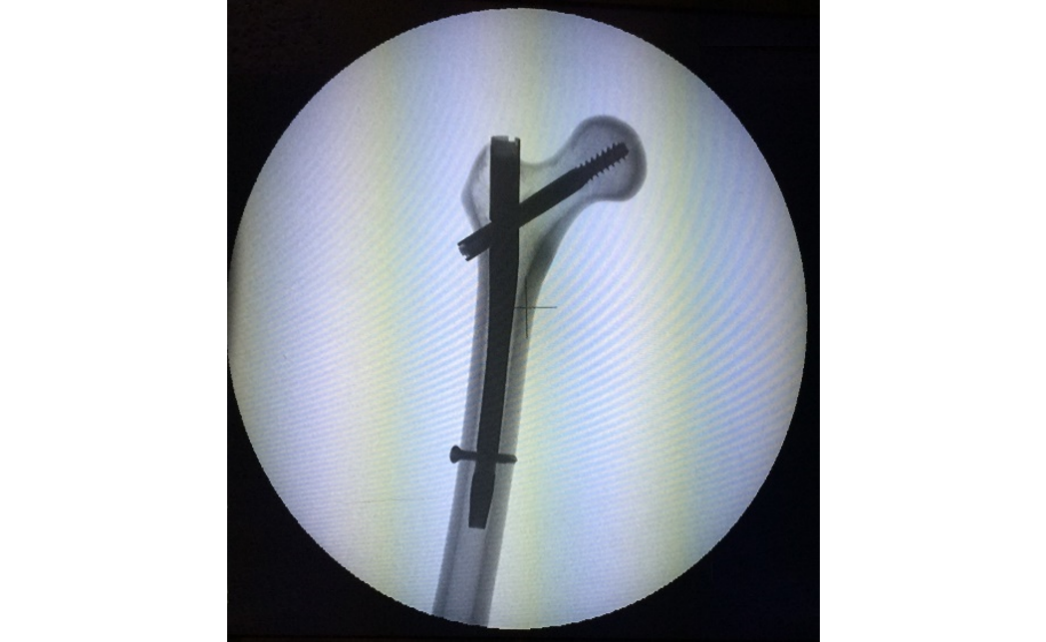
Instrumented Sawbone with Gamma3 nail.

**Figure 2:**
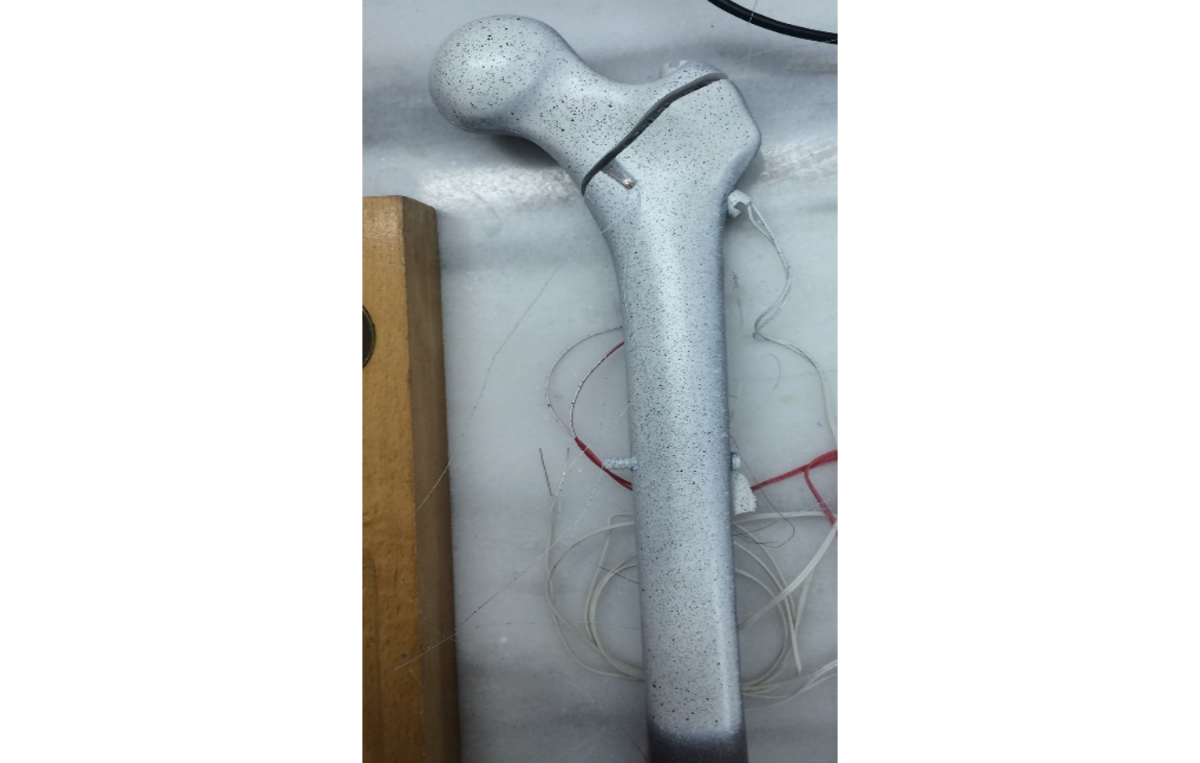
Instrumented stable intertrochanteric fracture with digital image correlation paint and strain gauges.

The instrumentation will be performed by GK in a standardized fashion on intact (prior to fracture creation) Sawbones under image intensifier to ensure a uniform implant position and TAD of the hip screw, which will be in the range of 10 to 20 mm (Figure 1). The Sawbones will then be uninstrumented, and the fractures will be created with the aid of a cutting guide to ensure identical fracture lines. Stable fractures will be created with a fracture line running 47° from the horizontal level exiting above the lesser trochanter, while the unstable fractures will have a wedge of bone removed contained the lesser trochanter. The Sawbones will then be reinstrumented and prepared for testing. A resin mold has been created that covers the distal femoral condyles and permits sufficient stabilization of the distal femur. The femur-resin mold block will be further stabilized by the use of a stainless-steel orthogonal holder and positioned at neutral position in the sagittal plane and 11° of adduction. Strain gauges will be applied on specific points of interest, namely the distal fragment including the calcar area and the distal fixation points, and a layer of matte white paint will be applied followed by black dots, thus creating a random speckle pattern (Figure 2).

#### Loading Configuration

The femurs will be fixed distally in resin in a steel block with neutral flexion-extension and 11° of adduction to simulate single leg stance [17]. The load will be transmitted by means of a steel plate to allow for rotation and translation as the distal femur is fixed. As the abductor insertion can be part of the fracture, the abductor pull will not be simulated to minimize confounding factors and excess motion at the fracture site. The position that will be studied is the single leg stance in the nonconsolidated fracture status [33].

**Figure 3:**
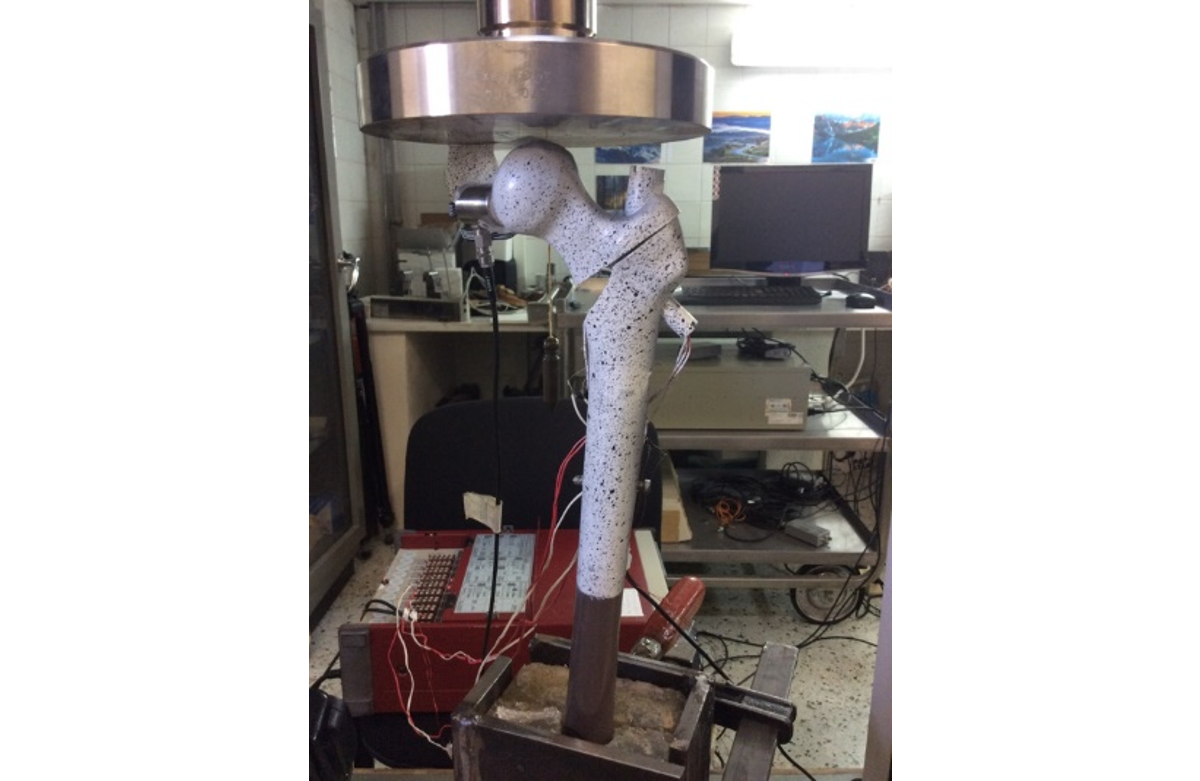
Loading configuration in unstable trochanteric fracture.

Mechanical testing will be undertaken in an MTS Insight 10 kN load frame (Testworks 4, MTS Systems Corp), and data will be retrieved with the aid of KFG Series strain gages (Kyowa Electronic Instruments Co Ltd) and a 3D image correlation system (3D-DIC, LIMESS Messtechnik und Software GmbH). The loading will include a 200 nt preload and relaxation followed by static loading until 2000 nt, which simulates the loads experienced by a hip during single leg stance (Figure 3). The implants will not be loaded to failure as static failure is not as clinically relevant as fatigue failure, which happens at submaximal loads after a high number of cycles.

#### Digital Image Correlation

The setup for digital image correlation entails two digital cameras placed so as to record the femoral head, neck, and proximal cortex from different angles [30]. All femurs will have a random but unique speckled pattern painted on them. As the load will be applied and deformation of the patterns will occur, they will be recorded throughout the loading process and consequently analyzed with the aid of specialized software (Aramis Professional, GOM). The differences between the patterns will allow the detailed mapping of the strain fields on the cortical bone.

#### Statistical Analysis

Statistical analysis will be performed using SPSS Statistics version 23 (IBM Corp). A Shapiro-Wilk test will be used to test for normality of distribution of the main results (ie, stress and construct stiffness). Homogeneity of variances between the groups will be checked with a Levene test. Significant differences between the 2 groups will be checked with paired samples *t* tests. Level of significance will be set to *P*=.05 for all statistical tests.

### Finite Element Analysis

The loading setting will be originally validated with the loading of an intact femur and comparison of the FEA of an intact femur. Consequently, FEA will be undertaken for stable and unstable fractures treated with DHS and gamma nail and a minimum of two experiments per scenario tested experimentally.

The FEA will be conducted using Ansys 16.0 (Ansys Inc). The femoral model to be used will be identical to the Sawbones as provided by Sawbones (digital models of the purchased Sawbones). The implants to be used will be designed by the use of 3D scanning and manual design using Solidworks 2016 (Dassault Systemes). Threads will not be used in the analysis to reduce computing requirements and the risk of abnormally high peak stresses on the thread tips. The optimal type of element as well as element size and meshing refinements will be decided based on convergence studies. However, at areas of great interest, such as the bone overlying the tip of the hip screw and the fracture area, small element sizes of under 2 mm will be used to optimize the accuracy of the results.

**Figure 4:**
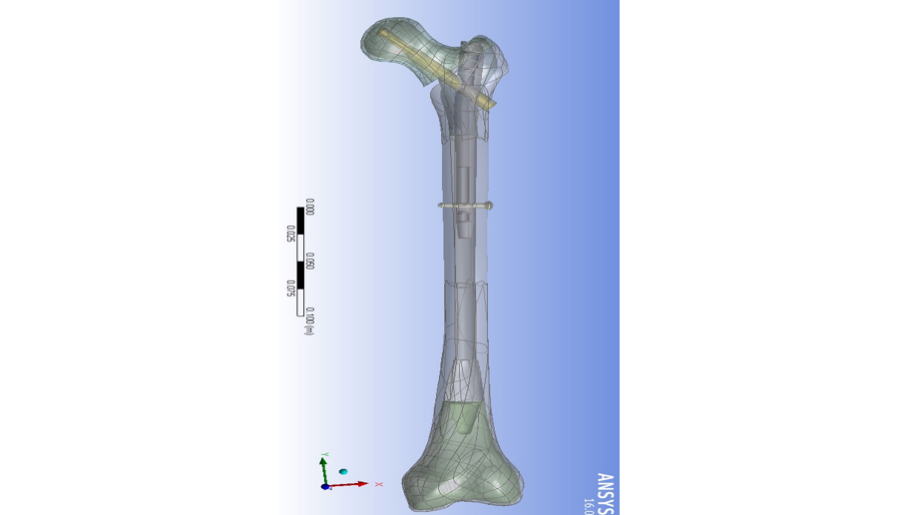
Unstable fracture finite element analysis model instrumented with Gamma3 (130/180/11).

The finite element (FE) model validation will be based on the experimental findings and will be done on two stages. First the intact Sawbone will be used to adjust for material properties attributed to cortical and cancellous bone and element size. Next the instrumented stable fractures will be used to refine meshing and element size and types, and finally the unstable fracture models will be used for fine-tuning of the FEA (Figure 4). After validation of the models, a series of fracture and reduction scenarios will be run to test our hypothesis. The effect of variable reduction angles and implant angles on the stresses incurring at the area of bone overlying the tip of the hip screw, the medial cancellous bone, and the implant and distal fixation sites will be analyzed. Additionally, the effect of distal locking static, dynamic, or no locking will be studied as well as the effect of various TAD and neck-shaft angle combinations (Textbox 1).

**Fracture-osteosynthesis scenarios to be studied.**

Unstable fractures treated with dynamic hip screw (DHS)

- Varus reduction, 130 plate, tip to apex distance (TAD)<25
- Varus reduction, 130 plate, TAD>25
- Varus reduction, 135 plate, TAD<25
- Varus reduction, 135 plate, TAD>25
- Varus reduction, 145 plate, TAD<25
- Varus reduction, 145 plate, TAD>25
- Anatomic reduction, 130 plate, TAD<25
- Anatomic reduction, 130 plate, TAD>25
- Anatomic reduction, 135 plate, TAD<25
- Anatomic reduction, 135 plate, TAD>25
- Anatomic reduction, 145 plate, TAD<25
- Anatomic reduction, 145 plate, TAD>25
- Valgus reduction, 130 plate, TAD<25
- Valgus reduction, 130 plate, TAD>25
- Valgus reduction, 135 plate, TAD<25
- Valgus reduction, 135 plate, TAD>25
- Valgus reduction, 145 plate, TAD<25
- Valgus reduction, 145 plate, TAD>25

Unstable fractures treated with gamma nail

- Varus reduction, 120 nail, TAD<25, static locking
- Varus reduction, 120 nail, TAD>25, static locking
- Varus reduction, 125 nail, TAD<25, static locking
- Varus reduction, 125 nail, TAD>25, static locking
- Varus reduction, 130 nail, TAD<25, static locking
- Varus reduction, 130 nail, TAD>25, static locking
- Varus reduction, 120 nail, TAD<25, dynamic locking
- Varus reduction, 120 nail, TAD>25, dynamic locking
- Varus reduction, 125 nail, TAD<25, dynamic locking
- Varus reduction, 125 nail, TAD>25, dynamic locking
- Varus reduction, 130 nail, TAD<25, dynamic locking
- Varus reduction, 130 nail, TAD>25, dynamic locking
- Varus reduction, 120 nail, TAD<25, unlocked
- Varus reduction, 120 nail, TAD>25, unlocked
- Varus reduction, 125 nail, TAD<25, unlocked
- Varus reduction, 125 nail, TAD>25, unlocked
- Varus reduction, 130 nail, TAD<25, unlocked
- Varus reduction, 130 nail, TAD>25, unlocked
- Anatomic reduction, 120 nail, TAD<25, static locking
- Anatomic reduction, 120 nail, TAD>25, static locking
- Anatomic reduction, 125 nail, TAD<25, static locking
- Anatomic reduction, 125 nail, TAD>25, static locking
- Anatomic reduction, 130 nail, TAD<25, static locking
- Anatomic reduction, 130 nail, TAD>25, static locking
- Anatomic reduction, 120 nail, TAD<25, dynamic locking
- Anatomic reduction, 120 nail, TAD>25, dynamic locking
- Anatomic reduction, 125 nail, TAD<25, dynamic locking
- Anatomic reduction, 125 nail, TAD>25, dynamic locking
- Anatomic reduction, 130 nail, TAD<25, dynamic locking
- Anatomic reduction, 130 nail, TAD>25, dynamic locking
- Anatomic reduction, 120 nail, TAD<25, unlocked
- Anatomic reduction, 120 nail, TAD>25, unlocked
- Anatomic reduction, 125 nail, TAD<25, unlocked
- Anatomic reduction, 125 nail, TAD>25, unlocked
- Anatomic reduction, 130 nail, TAD<25, unlocked
- Anatomic reduction, 130 nail, TAD>25, unlocked
- Valgus reduction, 120 nail, TAD<25, static locking
- Valgus reduction, 120 nail, TAD>25, static locking
- Valgus reduction, 125 nail, TAD<25, static locking
- Valgus reduction, 125 nail, TAD>25, static locking
- Valgus reduction, 130 nail, TAD<25, static locking
- Valgus reduction, 130 nail, TAD>25, static locking
- Valgus reduction, 120 nail, TAD<25, dynamic locking
- Valgus reduction, 120 nail, TAD>25, dynamic locking
- Valgus reduction, 125 nail, TAD<25, dynamic locking
- Valgus reduction, 125 nail, TAD>25, dynamic locking
- Valgus reduction, 130 nail, TAD<25, dynamic locking
- Valgus reduction, 130 nail, TAD>25, dynamic locking
- Valgus reduction, 120 nail, TAD<25, unlocked
- Valgus reduction, 120 nail, TAD>25, unlocked
- Valgus reduction, 125 nail, TAD<25, unlocked
- Valgus reduction, 125 nail, TAD>25, unlocked
- Valgus reduction, 130 nail, TAD<25, unlocked
- Valgus reduction, 130 nail, TAD>25, unlocked

### Design of the New Implant (Hybrid Nail)

**Figure 5:**
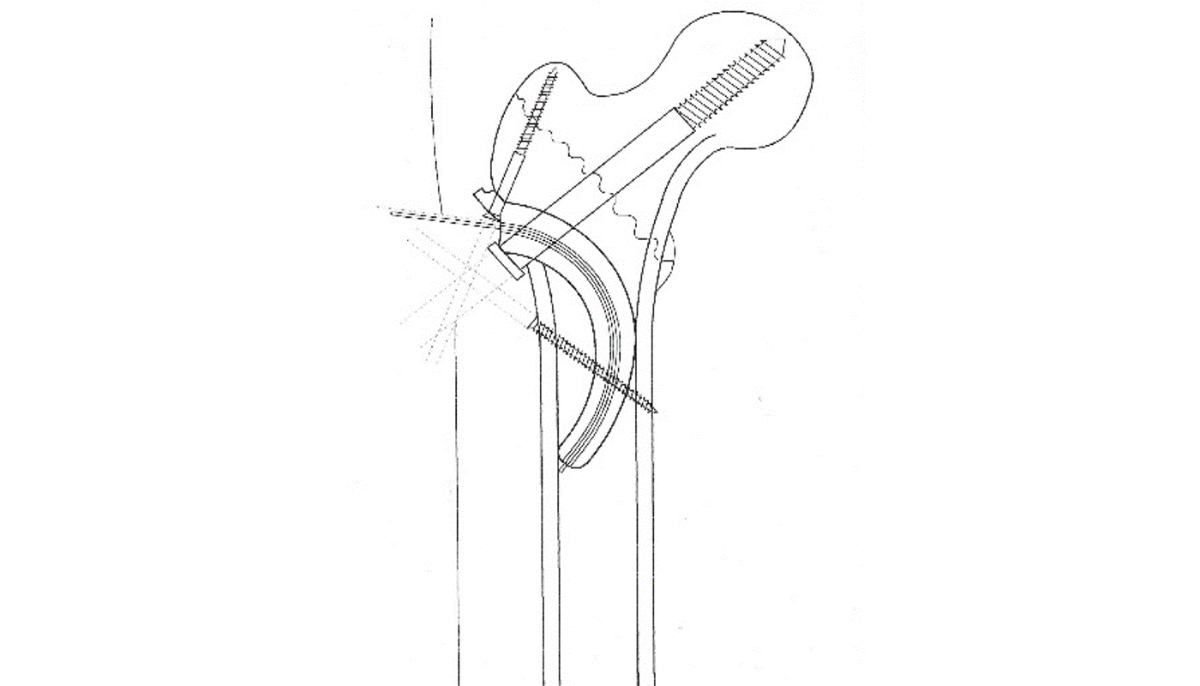
The proposed hybrid nail.

The new hybrid nail combines properties of both techniques. Using a small entry point beneath the greater trochanter, a semicircular solid nail of different length and diameter is introduced inside the medullary canal and impacted in the cortex, thus avoiding distal locking. The latter can be used if extra stability is needed. From the same entry point, the sliding hip screw can be inserted as well as a small trochanteric screw for unstable fracture patterns (Figure 5).

## Results

The DRIFT study will test the hypothesis that a valgus reduction is mechanically favorable in all fracture configurations, quantify the mechanical effect, and determine the optimal postreduction implant/bone geometry.

DRIFT also aims to quantify the mechanical effects of calcar and lateral wall integrity and would define the necessity to address them. DRIFT will study the effect of other variables on TAD and its cut-off point, with a working hypothesis that TAD is not independent from fracture characteristics, reduction angle, and screw position.

Also, DRIFT will test and quantify the effect of eccentric screw positioning on fracture-implant mechanics on a multiplanar gait-based model and will test the hypothesis on FEA models aiming to prove that distal locking is not necessary in most cases of stable fracture types.

In DRIFT, finally, the stress and strain fields, which will be derived by the FE method, will be used to predict failure of existing tools under static loading conditions. Moreover, the design optimization modules that appear in the existing commercial FE codes will be used for the numerical design of the novel implant with respect to its mechanical performance.

## Discussion

### Summary

Despite extensive literature on the various prognostic factors of mechanical failure in the osteosynthesis of pertrochanteric fractures, the effect of reduction angle has been understudied and the implant angle has not received significant attention. The main prognostic factor recognized is the TAD where a value of over 25 mm is being considered an independent predictive factor for failure. In a recent study from Bojan et al [28], the typical cut-out complication was represented by an unstable fracture type involving the trochanteric or cervical regions or both as well as nonanatomical reduction or nonoptimal screw position. The authors suggested that in order to reduce the risk of a cut-out it is important to achieve both anatomical reduction and optimal lag screw position as these are the only two factors that can be controlled by the surgeon. In general, the factors affecting optimal treatment of trochanteric fractures can be divided in two categories: fracture pattern (varus or valgus reduction, lesser trochanter integrity, and lateral wall integrity) and optimal implant positioning (TAD, position of lag screw, and distal locking).

### Fracture Pattern

#### Valgus/Varus Reduction

A higher postreduction neck shaft angle intuitively results in a greater fracture compression force vector and a subsequently lower ratio of force causing shear at the screw-bone interface. Parker [34] originally suggested valgus reduction to prevent cut-out. Despite biomechanical data [35,36] suggesting that a valgus postreduction angle would facilitate initiation of hip screw sliding in fractures treated by both sliding hip screw and intramedullary devices and therefore increase interfragmentary compression, this has not been confirmed in vivo by most relevant studies. In their randomized prospective clinical study, Pajarinen et al [14] noticed a postoperative decrease in the neck-shaft angle of operatively treated unstable pertrochanteric femoral fractures. A reduction in slight valgus was advocated for the unstable fractures to normalize the posthealing anatomic outcome. Recently, a retrospective study by Andruskow et al [37] in 235 patients found a trend that did not reach statistical significance (*P*=.19) for patients with a postoperative valgus neck-shaft angle of 5° to 10° to have a smaller chance of developing a cut-out. On the other hand, Pervez et al [38] compared 23 cases of cut-out with 77 cases of uneventful fracture healing and suggested that a varus reduction results in an increased incidence of cut-out.

#### Lesser Trochanter (Calcar) Integrity

The integrity of the lesser trochanter has been implicated as a potential prognostic factor of cut-out. Eberle et al [39] showed that the lack of calcar support effectively makes the implant a load-bearing device, placing more stresses on the implant in their biomechanical study using an FE model. Bojan et al [28] retrospectively reviewed 3066 cases of pertrochanteric fractures treated with trochanteric nails and found a statistically significant increase in the incidence of cut-out among patients with unstable complex fractures. The restoration of the posteromedial calcar fragments is considered a key point to achieve stable fracture fixation; on the contrary, failure to address calcar integrity can lead to higher mechanical failure rates as has been shown in two clinical studies using sliding hip screws [40,41].

#### Lateral Wall Integrity

Gotfried [42] retrospectively analyzed 24 patients with mechanical failure of a sliding hip screw intertrochanteric fracture fixation due to excessive fracture collapse. There was a fracture of the lateral wall in all cases, and this was associated with an increased risk for mechanical failure. In fact, loss of lateral wall integrity is considered a relative contraindication for the use of a sliding hip screw device. To tackle these challenging fractures, Gupta et al [43], in a series of 74 patients, had good results with the use of trochanteric stabilizing plates in patients with lateral wall fracture. The small sample size and lack of control group, however, limit the power of the study. Babst et al [44] had similarly good results in their prospective clinical study using trochanteric stabilizing plates. The use of proximal femoral nails has been advocated in these fractures; however, this has not been clinically proven in randomized controlled studies, and most relevant studies have methodological limitations or are of lower level evidence, as shown by Kregor et al [45] in their review paper.

### Optimal Implant Positioning

#### Tip to Apex Distance

**Figure 6:**
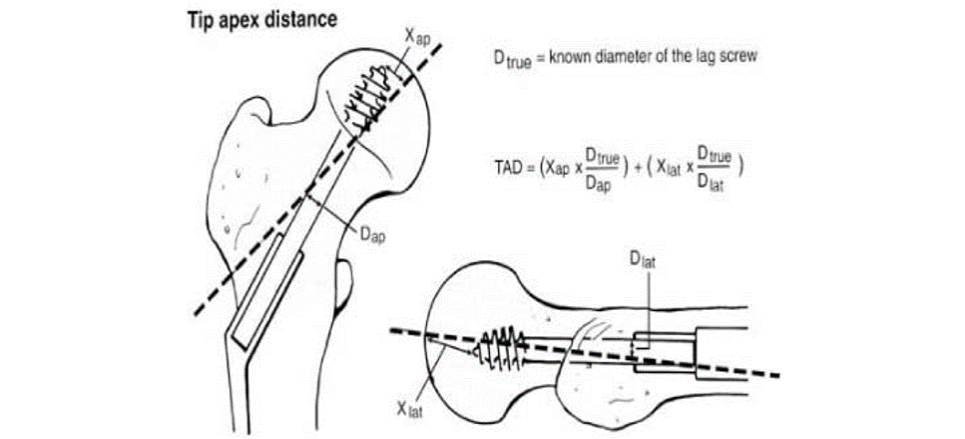
Calculation of tip to apex distance in the anteroposterior and lateral view.

TAD was defined in the original work of Baumgaertner et al [21] as the sum of the distance from the tip of the screw to the apex of the femoral head in the anteroposterior and lateral views, after controlling for magnification (Figure 6).

TAD is being widely considered [37,40,46-48] as the only independent predictive factor of cut-out. Additionally, the awareness of TAD among surgeons was shown to reduce mechanical failure [49]. A biomechanical cadaveric study by Kane et al [50] challenged the notion that a TAD greater than 24 mm leads to increased cut-out rates regardless of screw position and found that central inferior position of the hip screw was at least as biomechanically stable as the center-center position although the TAD was greater than 25 mm. Hsueh et al [51], in a retrospective evaluation of 937 patients treated with DHS (135° angle), suggested placing the lag screw in the middle/middle or inferior/middle position with appropriate TAD (<15 mm).

#### Position of Lag Screw

In most reports, cut-out has been evaluated on two-dimensional radiographs, showing varus collapse of the femoral head and superior cut-out of the lag screw. The biomechanical studies are almost exclusively based on axial static or dynamic loading in only one plain. Ehmke et al [52], in their study, applied multiplane loading of pertrochanteric fracture models and suggested that cut-out occurs due to combined axial loads and rotational moments as in normal walking. Lenich et al [53] advocated a central position of the hip screw or blade as the optimal position to minimize rotational forces on the femoral head.

#### Distal Locking

Distal locking in pertrochanteric fractures nailing is the standard of practice. The necessity of distal interlocking screws in stable intertrochanteric fractures has been biomechanically challenged in a study by Rosenblum et al [54]. This study, however, used the first-generation gamma nail, and the results cannot be safely applied to newer generation trochanteric nails that tend to be less stiff than the original. Skála-Rosenbaum et al [55], in their prospective clinical study, compared stable trochanteric fractures treated either with distal dynamic locking (44 cases) or without locking (77 cases) and found no difference in terms of time to healing, functional results, and complications. The authors proposed that distal locking is unnecessary in stable intertrochanteric fractures. Finally, Lobo-Escolar et al [46], in their case controlled clinical study, found positive correlation between distal static locking and cut-out, but these results did not reach statistical significance. Currently, use of the FE methods to study trochanteric fractures has been confined to simple static stress analyses of existing tools aiming to recognize the critical stresses and strains in the bone.

### Strengths and Limitations

Strengths of this study include the use of uniform material and digital resources, thus negating the need for adjustments and variability between the biomechanical testing and subsequent FEA validation. This is, to the best of our knowledge, the first biomechanical study to evaluate the effect of both reduction angle and implant angle in fracture treatment.

The main weakness of the study is the static nature of the loading used. Despite the fact that in clinical practice failure is a dynamic effect, this study tests the initial loading characteristics of various fracture configurations, a necessary prerequisite for any future cyclic loading studies.

### Conclusion

Despite recent advances, cut-out remains the most common and devastating mechanical complication of intertrochanteric fracture treatment. Taking into consideration the increased health risks related to the treatment of this complication alongside the increased hospitalization and health care costs in the setting of an aging European population, the need to improve treatment outcomes of these fractures is evident. This entails both enhancing our understanding of the prognostic factors of cut-out and improving all aspects of intertrochanteric fracture treatment. The optimization of the biomechanical behavior of the fracture-osteosynthesis model by the application of the ideal reduction angle and implant is expected to have a positive effect on the rate of mechanical failure and, subsequently, the healing rates, morbidity, and mortality in this fragile patient group.

## Abbreviations

CMN: cephalomedullary nails
DHS: dynamic hip screw
DRIFT: Design of Improved Intertrochanteric Fracture Treatment
FE: finite element
FEA: finite element analysis
TAD: tip to apex distance
